# Glutathione *S*-Transferases in the Biosynthesis of Sulfur-Containing Secondary Metabolites in Brassicaceae Plants

**DOI:** 10.3389/fpls.2018.01639

**Published:** 2018-11-13

**Authors:** Paweł Czerniawski, Paweł Bednarek

**Affiliations:** Institute of Bioorganic Chemistry, Polish Academy of Sciences, Poznań, Poland

**Keywords:** glutathione, glutathione *S*-transferase (GST), glucosinolate, sulfur-containing phytoalexin, Brassicaceae

## Abstract

Plants in the Brassicaceae family have evolved the capacity to produce numerous unique and structurally diverse sulfur-containing secondary metabolites, including constitutively present thio-glucosides, also known as glucosinolates, and indole-type phytoalexins, which are induced upon pathogen recognition. Studies on the glucosinolate and phytoalexin biosynthetic pathways in the model plant *Arabidopsis thaliana* have shown that glutathione donates the sulfur atoms that are present in these compounds, and this further suggests that specialized glutathione *S*-transferases (GSTs) are involved in the biosynthesis of glucosinolates and sulfur-containing phytoalexins. In addition, experimental evidence has shown that GSTs also participate in glucosinolate catabolism. Several candidate GSTs have been suggested based on co-expression analysis, however, the function of only a few of these enzymes have been validated by enzymatic assays or with phenotypes of respective mutant plants. Thus, it remains to be determined whether biosynthesis of sulfur-containing metabolites in Brassicaceae plants requires specific or nonspecific GSTs.

## Introduction

Glutathione *S*-transferases (GSTs) constitute a family of multifunctional enzymes that catalyze the nucleophilic attack of the sulfur atom of the tripeptide glutathione (GSH) on electrophilic centers of low-molecular weight compounds (Dixon and Edwards, 2010; Labrou et al., 2015). GSTs were identified as stress response proteins that accumulated in response to biotic and abiotic stimuli. Many studies on plant GSTs have focused on their role in xenobiotic detoxification. In addition, some GSTs have been implicated in plant secondary metabolism, particularly in the formation of natural products containing carbon-sulfur bonds, including the sulfur-containing phytochemicals characteristic of Brassicaceae species (Dixon et al., 2010; Sonderby et al., 2010; Pedras et al., 2011; Bednarek, 2012; Dunbar et al., 2017).

## Conjugation of GSH is Required for the Biosynthesis of Glucosinolates

**FIGURE 1:**
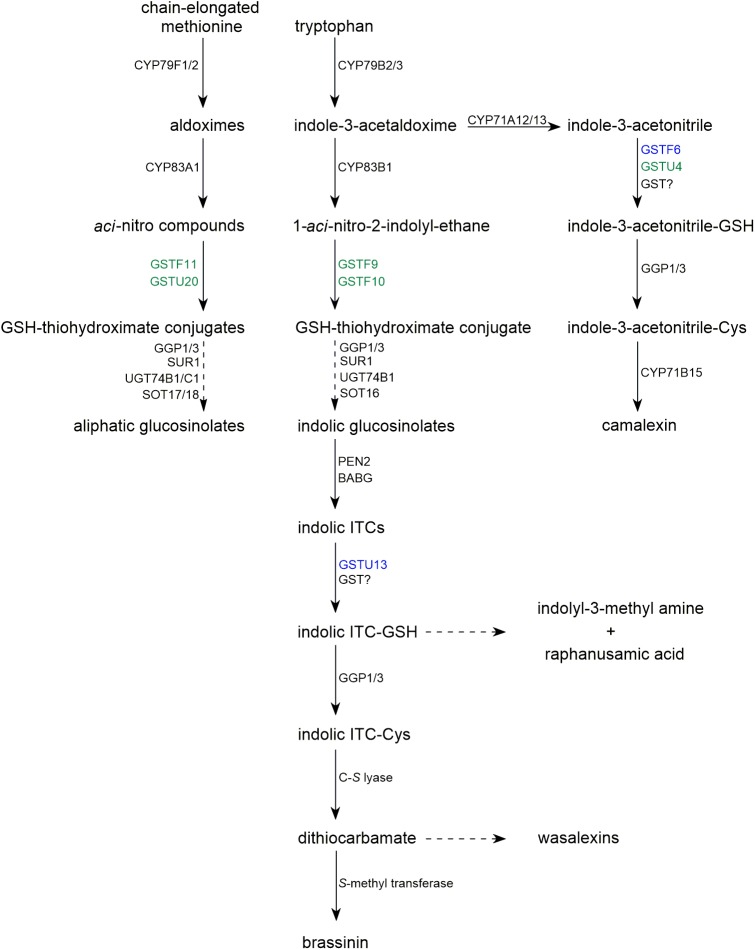
Biosynthetic pathways of glucosinolates and selected sulfur-containing phytoalexins occurring in Brassicaceae. Dashed arrows represent multistage processes. GSTs with function confirmed with mutant phenotype are indicated in blue. GSTs with contribution proposed based on co-expression analysis are indicated in green.

Glucosinolates are sulfur-containing secondary metabolites produced by plants of the Brassicales order, and their core structure contains a β-D-thioglucose moiety connected to a sulfonated aldoxime and a variable side chain derived from amino acids, such as tryptophan, tyrosine, and methionine (Halkier and Gershenzon, 2006). The first two steps of glucosinolate biosynthesis are catalyzed by specific isoforms of CYP79 and CYP83 cytochrome P450 monooxygenases, which convert precursor amino acids to aldoximes and then to *aci*-nitro compounds. It has been postulated that these intermediates can react with an alkylthiol to form conjugates that can be converted to glucosinolates by the sequential activities of C-*S* lyase (SUR1), glucosyltransferases (UGTs), and sulfotransferases (SOTs) (Figure 1; Sonderby et al., 2010). Decreased glucosinolate accumulation in the *phytoalexin deficient 2* (*pad2*) mutant, which has a reduced GSH biosynthesis rate, suggested that GSH is the alkylthiol that conjugates with the products of CYP83 activity (Parisy et al., 2007; Schlaeppi et al., 2008). In line with this hypothesis, upon engineering benzyl glucosinolate biosynthesis in *Nicotiana benthamiana*, it was found that expression of CYP79A2 and CYP83B1 led to an accumulation of *S*-(phenylacetohydroximoyl)-GSH, the predicted GSH conjugate (Geu-Flores et al., 2009). Introduction of SUR1, UGT74B1, and SOT18 into the engineered *N. benthamiana* line led to low level production of benzyl glucosinolate, but did not significantly reduce the *S*-(phenylacetohydroximoyl)-GSH level suggesting that this intermediate is not a substrate of SUR1. This was confirmed by additional expression of γ-glutamyl peptidase 1 (GGP1) or GGP3, enzymes cleaves γ-Glu from GSH conjugates, which resulted in depletion of the *S*-(phenylacetohydroximoyl)-GSH intermediate along with a significant increase in the rate of benzyl glucosinolate production in transgenic *N. benthamiana* (Geu-Flores et al., 2009, 2011). In addition, glucosinolate levels decreased and levels of the corresponding GSH-containing intermediates increased in an Arabidopsis *ggp1 ggp3* double mutant (Geu-Flores et al., 2011).

**FIGURE 2:**
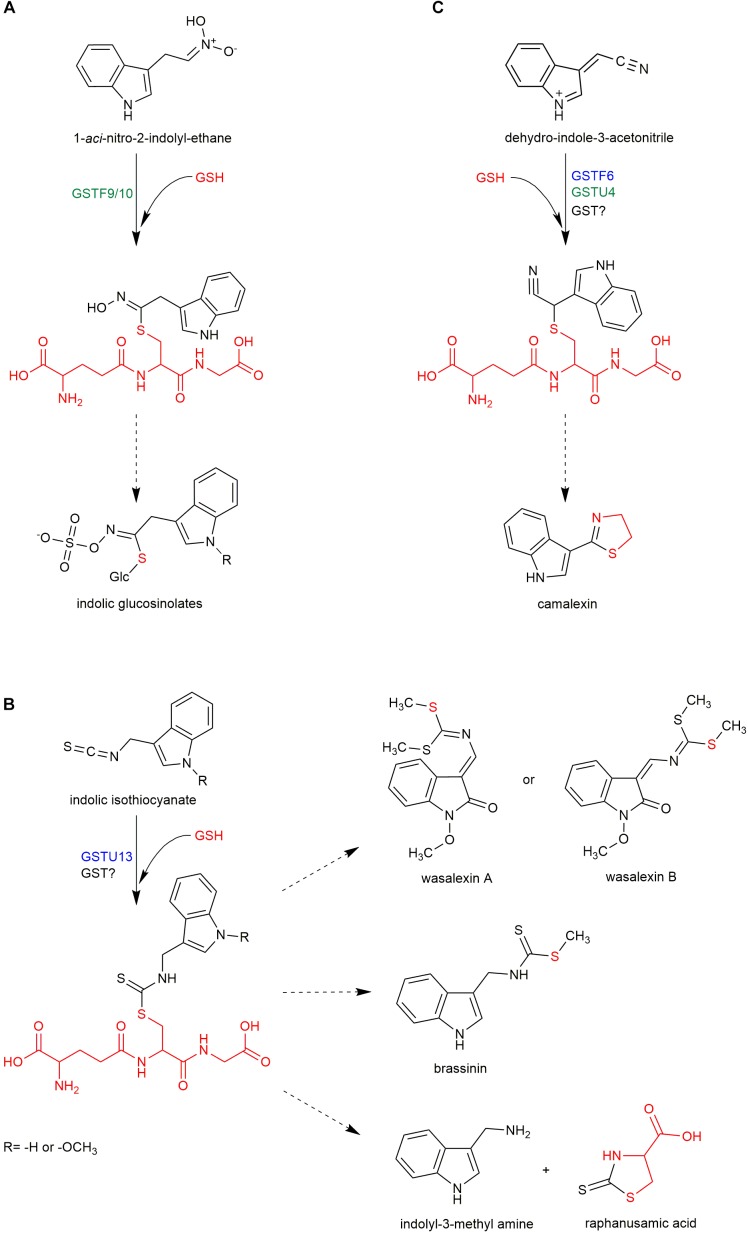
Proposed involvement of GSTs in biosynthesis of indolic glucosinolates **(A)**, camalexin **(C)**, and other sulfur-containing phytoalexins **(B)**. Dashed arrows represent multistage processes. Red color indicates glutathione and its fragments in structures of respective sulfur-containing metabolites. GSTs with function confirmed with mutant phenotype are indicated in blue. GSTs with contribution proposed based on co-expression analysis are indicated in green.

Collectively, these findings confirmed that GSH-conjugates are glucosinolate biosynthetic intermediates and raised the question of whether conjugation of GSH with products of CYP83 activity requires specific enzymatic activity. Candidate GSTs involved in this process have been proposed based on their co-expression with glucosinolate biosynthesis enzymes and on an analysis of metabolic and gene expression profiles of quantitative trait loci (Hirai et al., 2005; Wentzell et al., 2007; Hirai, 2009). It has been suggested that GSTF11 and GSTU20 are involved in aliphatic glucosinolate (AG) biosynthesis and that GSTF9 and GSTF10 contribute to indolic glucosinolate (IG) formation (Figures 1,2A). In addition, transcriptome analyses of Arabidopsis *myb28* knock-out and *MYB28*-overexpressing cell cultures showed that *GSTF11* and *GSTU20* expression is regulated by the MYB28 transcription factor, which controls the AG biosynthetic pathway (Hirai et al., 2007).

However, despite these correlations, no GST function in glucosinolate biosynthesis has yet been validated experimentally. For instance, successful engineering of benzyl glucosinolate or glucoraphanin (an AG) biosynthesis in *N. benthamiana* did not require any Arabidopsis GSTs (Geu-Flores et al., 2009; Mikkelsen et al., 2010). Moreover, introduction of GSTF11 increased the efficiency of glucoraphanin production by only 20% (Mikkelsen et al., 2010). Similarly, expression of Arabidopsis IG biosynthetic genes in yeast (*Saccharomyces cerevisiae*) showed that GSTF9 and GSTF10 are dispensable for conjugation of the product of CYP83B1 activity with GSH in this microorganism. Additional introduction of GSTF9 in the engineered yeast strain boosted the level of glucosinolate by only 25% (Mikkelsen et al., 2012). These results suggest that GSH conjugation in glucosinolate biosynthesis can occur spontaneously without GST activity or that tested GSTs are not specific for glucosinolate biosynthesis and can be replaced by GSTs from other organisms. However, overexpression of Arabidopsis enzymes in *N. benthamiana* or in yeast obscures their native temporal and spatial accumulation patterns. Opposite, fluorophore-tagged glucosinolate biosynthetic enzymes, including CYP83 monooxygenases that produce putative GST substrates, localized to specific tissues and cell types when expressed under their native promoters in Arabidopsis (Nintemann et al., 2018). Thus, it is likely that GSTs involved in glucosinolate biosynthesis are not specific with regards to their substrate preference or catalytic properties, but can be specific with regards to their localization, which can be not observed in glucosinolate-engineered strains.

In addition to the missing functional validation, experiments have suggested that the GSTs that have been proposed to contribute to glucosinolate biosynthesis may have alternative *in planta* functions. For instance, in a yeast two-hybrid screen, GSTU20 interacted with Far-Red Insensitive 219, a jasmonate-conjugating enzyme linked to phytochrome signaling, and a partial loss of GSTU20 function resulted in hyposensitivity to continuous far-red light. Moreover, under the same condition *GSTU20* was differentially expressed in *suppressor of phytochrome A-105 1* and *constitutive photomorphogenic 1* mutant plants (Chen et al., 2007). To explain these phenotypes, it has been hypothesized that GSTU20 can bind, stabilize, or transport jasmonic acid or its derivatives within the cell.

Another yeast two-hybrid screen indicated that GSTF10 interacts with Brassinosteroid Insensitive 1 (BAK1), a leucine-rich repeat receptor-like kinase involved in brassinosteroid signaling and plant defense (Ryu et al., 2009). RNA interference (RNAi)-mediated down-regulation of *GSTF10* and *GSTF9* expression led to a more compact rosette shape, which is similar to the phenotype of weak *bak1* mutant alleles. However, plants that underexpressed (via RNAi) or overexpressed *GSTF10* showed wild type (WT)-like growth in the presence of brassinolide (a brassinosteroid) or brassinazole (an inhibitor of brassinosteroid biosynthesis), thus GSTF10 is probably not involved in brassinosteroid signaling. In addition to the compact rosette phenotype, *GSTF10/9* RNAi plants had higher anthocyanin levels and a lower tolerance for NaCl or *N*-acetylcysteine, a pharmacological reagent that scavenges free radicals (Ryu et al., 2009). Similar to the RNAi line, a *gstf9* mutant had a lower tolerance for NaCl and was defective in redox homeostasis (Horváth et al., 2015). It has also been shown that GSTF9 is induced in response to the gravity persistent signal (GPS), and *gstf9* mutants displayed defective GPS responses in inflorescence stems, as well as in root skewing, waving, and curvature (Schenck et al., 2013). Collectively, these findings suggest that GSTF9 and GSTF10 contribute to redox homeostasis and responses to environmental stimuli, but it is unclear whether these putative functions depend on glucosinolate biosynthesis.

## GSTs are Important for Glucosinolate Metabolism

Specific β-thioglucosidases, known as myrosinases, and glucosinolates constitute a binary defense system against generalist insects and pathogens (Hopkins et al., 2009; Pastorczyk and Bednarek, 2016). Upon tissue damage or in response to environmental stimuli, glucosinolates can be hydrolyzed by myrosinases leading to the formation of unstable aglycones. Based on their side chain structure and the presence of specifier proteins, these aglycones can rearrange into different end products, including highly chemically reactive and biologically active isothiocyanates (ITCs), which can be harmful for the host plant (Wittstock et al., 2016). It has been shown that exogenous ITC application has negative effects on Arabidopsis growth (Hara et al., 2010; Urbancsok et al., 2017). Notably, Arabidopsis GSH-deficient mutants have been shown to be more susceptible to ITCs than WT plants suggesting that deactivation of ITCs *in planta* requires their conjugation with GSH (Urbancsok et al., 2018). As indicted by experimental evidence this reaction is spontaneous, but its efficiency can be significantly enhanced with GST-mediated catalysis, and leads to the formation of dithiocarbamate-type ITC-GSH adducts (Zhang et al., 1995). Enzymatic studies demonstrated that many Arabidopsis GSTs process benzyl-ITC, which is a model ITC used in *in vitro* enzyme assays (Wagner et al., 2002; Dixon et al., 2009). Moreover, in Arabidopsis, it has been shown that some GST genes are induced in response to external ITC application (Hara et al., 2010; Øverby et al., 2015). Overall, these results indicate that GSTs function in the detoxification of glucosinolate-derived ITCs in Brassicales plants.

In addition to its role in ITC detoxification, conjugation with GSH can lead to the formation of novel products with important roles in plant fitness. During the immune response in Arabidopsis, Penetration 2 myrosinase (PEN2) metabolizes IGs to several end products, including indol-3-yl methyl amine (I3A), raphanusamic acid (RA), and 4-*O*-β-glucosyl-indol-3-ylformamide (4OGlcI3F) (Figure 1; Bednarek et al., 2009; Lu et al., 2015). The reduced accumulation of these metabolites in GSH-deficient *pad2* plants indicates that their formation is GSH dependent (Bednarek et al., 2009; Piślewska-Bednarek et al., 2018). In addition, the structures of I3A and RA suggest that they are derived from a dithiocarbamate-type adduct formed from indol-3-ylmethyl-ITC (I3-ITC), a product of indol-3-ylmethyl glucosinolate (I3G) hydrolysis (Figure 2B). However, in contrast to aliphatic- or benzyl-ITCs, indolic ITCs are highly unstable, and their spontaneous conjugation with GSH is preceded by a release of a thiocyanate ion leading to products different from dithiocarbamates (Kim et al., 2008; Agerbirk et al., 2009). Thus, the formation of I3A and RA most likely requires a GST that can efficiently conjugate GSH with the labile I3-ITC formed by PEN2 myrosinase, and gene co-expression analysis pointed to GSTU13 as a candidate for this function (Piślewska-Bednarek et al., 2018). This selection was additionally supported by *in vitro* enzymatic assays, which indicated that among 35 tested Arabidopsis GSTs GSTU13 together with GSTU4 and GSTU6 had not only the highest activity against benzyl-ITC, but also the highest specificity toward this compound as compared with the other tested substrates (Wagner et al., 2002; Dixon et al., 2009). The reduced accumulation of I3A, RA, and 4OGlcI3F observed in *gstu13* mutant plants confirms that GSTU13 is involved in biosynthesis of these compounds. Moreover, an analysis of the susceptibility of *pen2* and *gstu13* single and double mutants to selected fungal pathogens suggested that PEN2 and GSTU13 are part of the same immune pathway (Piślewska-Bednarek et al., 2018). Because PEN2, which localizes to the mitochondrial membranes, is actively delivered with a subpopulation of mitochondria to pathogen contact sites (Fuchs et al., 2016), in addition to its substrate specificity, spatial and temporal localization may also be critical for GSTU13 function.

## GSTs Contribute to Phytoalexin Biosynthesis

Apart from glucosinolates, Brassicaceae plants produce another group of sulfur-containing metabolites known as Brassicaceae phytoalexins. In general, phytoalexins are highly diverse, low molecular weight antimicrobial compounds that are produced in plants in response to infection. Phytoalexins produced by Brassicaceae plants are usually composed of an indole core and a side chain with one or two sulfur atoms (Pedras et al., 2011). Interestingly, it has been shown that biosynthesis of some indolic phytoalexins, including brassinin, is tightly linked with IG biosynthesis and metabolism (Figure 1). Brassinin is a phytoalexin produced by *Brassica* species that consists of an indole ring conjugated with an *S*-methylated dithiocarbamate group (Figure 2B). Upon application of benzyl-ITC to the roots of turnip plants (*Brassica campestris* ssp. *rapa*), a benzyl-type structural analog of brassinin was formed indicating that brassinin and related metabolites can be produced from IGs via corresponding ITCs (Monde et al., 1994). Similarly, upon application of labeled I3G to the leaves of salt cress (*Thellungiella salsuginea*), the label was incorporated into wasalexins, which are structurally related to brassinin (Figure 2B; Pedras et al., 2010). These results confirmed that IGs may serve as precursors to some Brassicaceae phytoalexins and raised the question of whether myrosinases are involved in the biosynthesis of these compounds. Transcriptome analysis of *Brassica rapa* combined with a comparative genomic approach to eliminate genes with direct orthologs in Arabidopsis, which does not produce brassinin, led to the identification of two Brassinin-Associated β-Glucosidases (BABGs), putative myrosinases that may hydrolyze IGs during biosynthesis of this phytoalexin (Klein and Sattely, 2017). Engineered expression of the IG pathway enzymes, the identified BABGs, and a dithiocarbamate *S*-methyltransferase, which catalyzes the last step in brassinin biosynthesis, in *N. bethamiana* resulted in accumulation of brassinin in transfected leaves confirming biosynthetic link between this compound and IGs. This link combined with the presence of the dithiocarbamate group in brassinin molecule suggests that biosynthesis of this phytoalexin involves the same I3-ITC-GSH adduct proposed as an intermediate in the PEN2 pathway (Figure 2B; Bednarek et al., 2009). This in turn raised the question of whether GSTs are involved in the conjugation of I3-ITC with GSH during brassinin biosynthesis. Brassinin was produced efficiently in transfected *N. bethamiana* leaves suggesting that the conjugation step can be catalyzed by *Br*GSTF9, which was included in the engineered IG pathway, or by nonspecific GSTs from *N. benthamiana* (Klein and Sattely, 2017). However, a relatively high level of indole-3-carbinol, an I3-ITC degradation product, also accumulated in the engineered *N. benthamiana* suggesting that *Br*GSTF9 or non-specific GSTs are insufficient to conjugate unstable I3-ITC with GSH efficiently, thus a specific GST may be involved in brassinin biosynthesis. Unfortunately, transcriptome analysis did not identify a unique *B. rapa* GST that was induced upon pathogen inoculation (Klein and Sattely, 2017).

The only identified sulfur-containing phytoalexin in Arabidopsis is camalexin, and production of this compound is dependent on sulfate nutritional status (Kruse et al., 2012). Reduced accumulation of camalexin in *pad2* mutant plants suggested that GSH is the precursor to the thiazole ring present in its structure (Figure 2C; Parisy et al., 2007). Camalexin shares the first biosynthetic step, conversion of tryptophan to indole-3-acetaldoxime by CYP79B2/3 enzymes, with IGs. In the next step, indole-3-acetaldoxime is converted by CYP71A12 and CYP71A13 to indole-3-acetonitrile (IAN), and then a conjugate of GSH and IAN (GS-IAN) is formed as indicated by enhanced accumulation of GS-IAN in a double mutant line depleted of GGP1 and GGP3, which cleave γ-Glu from this intermediate (Figure 1; Geu-Flores et al., 2011; Klein et al., 2013; Müller et al., 2015). However, despite the identification of GGPs as enzymes processing GS-IAN the nature of the substrate that reacts with GSH to form this conjugate remains obscure. Geu-Flores et al. (2011) suggested that an unknown enzyme activates IAN before conjugation with GSH. *In vitro* assays showed that CYP71A12/13 monooxygenases can play this role by further oxidizing IAN to α-hydroxy-IAN and to dehydro-IAN, which can react spontaneously with GSH (Figure 2C; Klein et al., 2013). Although the mechanism of *in planta* IAN activation remains unclear, it is likely that GSTs are involved in the subsequent biosynthetic step and respective enzymes have been searched for. It is known that camalexin biosynthesis is activated by the mitogen-activated protein kinase (MAPK) cascade, which includes MAPKK9 (Xu et al., 2008). Proteome analysis of constitutively active MAPKK9^DD^ transgenic plants showed that GSTF2, GSTF6, and GSTF7 accumulate to high levels during camalexin production (Su et al., 2011). To validate the putative function of these transferases, transgenic lines overexpressing GSTF2, GSTF6, or GSTF7 individually in the MAPKK9^DD^ background were generated, and a significant increase in camalexin production was observed in the GSTF6/MAPKK9^DD^ line indicating that GSTF6 contributes to camalexin biosynthesis. In addition, *gstf6* knock-out seedlings showed a slight but significant reduction in camalexin production suggesting that GSTF6 along with additional GSTs participate in biosynthesis of this phytoalexin (Su et al., 2011). A candidate GST involved in camalexin biosynthesis is GSTU4, which is co-expressed tightly with CYP71A13 and PAD3 (Piślewska-Bednarek et al., 2018), however, the function of this enzyme has not yet been evaluated experimentally. In contrast to the conclusions of Su et al. (2011), additional expression of *GSTF6*, in engineered *N. benthamiana* line expressing *CYP79B2*, *CYP71A13*, *GGP1*, and *PAD3* did not affect camalexin accumulation indicating that enzymatic catalysis is not required for GSH conjugation during biosynthesis of this phytoalexin or that *N. benthamiana* GSTs can replace GSTF6 (Møldrup et al., 2013). However, similar to the glucosinolate pathway, it is possible that GSTF6 specificity in camalexin biosynthesis results from its spatial and temporal expression pattern rather than from substrate specificity.

Despite the reported defect in camalexin accumulation in *gstf6* plants, experimental data suggests that GSTF6 plays alternative roles in anthocyanin biosynthesis and drought tolerance. Because *GSTF6* transcript levels were highly elevated in transgenic plants overexpressing *Production of Anthocyanin Pigment 1* (*PAP1*/*MYB75*), *GSTF6* expression appears to be regulated by the PAP1 transcription factor that controls anthocyanin biosynthesis (Tohge et al., 2005). In addition, *GSTF6*, also known as *Early Responsive to Dehydration 11*, was identified as a gene that is induced strongly in response to dehydration (Kiyosue et al., 1993), a condition that may induce anthocyanin biosynthesis (Nakabayashi et al., 2014). These findings suggest that GSTF6 may act redundantly with GSTF12, also known as Transparent Testa 19, which has been postulated to facilitate transport of anthocyanins and proanthocyanidins from the cytosol into the vacuole (Kitamura et al., 2004). However, in contrast to *gstf12*, *gstf6* mutant plants did not display any defects in anthocyanin accumulation (Wangwattana et al., 2008).

## Conclusion

Recent experimental evidence indicated that GSH-conjugates are intermediates in the biosynthesis of sulfur-containing secondary metabolites in Brassicaceae plants, thus there has been a search for the GSTs responsible for the formation of these intermediates. Several candidate GSTs have been identified based on co-expression with enzymes involved in the corresponding biosynthetic pathways. From those, so far only GSTF6 and GSTU13 have been shown to be required for the formation of the corresponding end products. Metabolic engineering of the Brassicaceae biosynthetic pathways in other organisms suggests that GSTs from Brassicaceae plants, with the possible exception of those involved in the conjugation of unstable indolic ITCs, are generalists rather than specific in their catalytic properties and substrate specificity. However, distinct spatial and temporal distributions of enzymes linked with IG biosynthesis and metabolism suggest that the specificities of GSTs involved in biosynthesis of sulfur-containing phytochemicals may result from their expression patterns and from their cellular and sub-cellular localizations. Therefore investigation of GSTs involved in the production of sulfur-containing phytochemicals from Brassicaceae should also address these aspects in a greater detail.

## Author Contributions

PC drafted the manuscript. PB supervised the writing, revised the manuscript, and prepared its final version.

## Conflict of Interest Statement

The authors declare that the research was conducted in the absence of any commercial or financial relationships that could be construed as a potential conflict of interest.
